# Inflammatory burden and cardiovascular risk stratification across psoriasis severity stages

**DOI:** 10.25122/jml-2026-0026

**Published:** 2026-03

**Authors:** Oana-Georgiana Văduva, Argyrios Periferakis, Alexandros Kanellos Mavrokefalos, Lamprini Troumpata, Aristodemos-Theodoros Periferakis, Bogdan Hategan, Priscila Mădălina Ologeanu, Roxana Elena Doncu, Liviu Mosoia Plaviciosu, Vlad Mihai Voiculescu, Călin Giurcăneanu

**Affiliations:** 1 Faculty of Medicine, Carol Davila University of Medicine and Pharmacy, Bucharest, Romania; 2 Akadimia of Ancient Greek and Traditional Chinese Medicine, Athens, Greece; 3 Elkyda, Research & Education Centre of Charismatheia, Athens, Greece; 4 Faculty of Biology, University of Bucharest, Bucharest, Romania; 5 Department of Gastroenterology, Emergency University Hospital, Bucharest, Romania; 6 Surgery Department, Carol Davila University Emergency Military Hospital, Bucharest, Romania; 7 Dermatology Department, Elias Emergency University Hospital, Bucharest, Romania

**Keywords:** psoriasis severity, inflammation markers, ESR, fibrinogen, CRP, neutrophils, cardiovascular risk assessment

## Abstract

Psoriasis is an autoimmune pathology with a pronounced inflammatory component, characterised by hallmark cutaneous symptoms and systemic inflammation that may involve multiple organ systems. Systemic inflammation can be quantified using nonspecific markers, such as erythrocyte sedimentation rate (ESR), fibrinogen levels, C-reactive protein (CRP), and neutrophil count. These markers usually correlate with disease severity. Since disease severity in psoriasis is quantified using the Psoriasis Area and Severity Index (PASI) score, we determined levels of these non-specific inflammatory markers in a convenience sample of patients with psoriasis. We attempted to correlate them with disease severity. There was a significant positive association between ESR levels and disease severity; similarly, fibrinogen was usually elevated in patients with more severe disease compared to those with milder disease, but remained within normal limits. Higher PASI scores were also associated with more severe disease. A non-statistically significant increase in neutrophil counts was observed in patients with more severe forms. Patients with more severe forms of the disease were more likely to have cardiovascular (CV) risk, and, based on our predictive model, increases in PASI score resulted in a quantifiable increase in CV risk. Therefore, taking our results into account, we propose that non-specific inflammation markers can be used to monitor disease severity and progression, and perhaps, response to therapy, and that careful monitoring for adverse CV events may be required in patients with severe psoriasis.

## Introduction

Over the last decades, the incidence of autoimmune diseases has increased; one of the most prominent is psoriasis, an autoimmune disease of complex aetiology and pathogenesis. The incidence and prevalence of psoriasis vary geographically and by population, but it is estimated that about 120 million people worldwide are affected by this disease [1]. The average prevalence, at least in adults, has been estimated to be up to 8% [2]. Even though there is a genetic predisposition [3], genetic determinism cannot account for all the detected cases [4].

Although it has different manifestations of varying severity, its most important, from the perspective of patient quality of life, are the cutaneous manifestations [5-8]. Despite cutaneous manifestations being in the spotlight, both for treatment and assessment, psoriasis is a systemic disease and may be associated with other comorbidities in many patients [9,10]. The inflammatory component of the disease is rather obvious in the cutaneous manifestations, and, oftentimes, the triggers themselves are factors and conditions leading to the release of pro-inflammatory factors, i.e., mechanical stress [11,12], psychological stress [13,14], infections [15,16], and gut microbiota dysbiosis [17,18]. Regarding mechanical stress in particular, it is known that psoriatic lesions usually occur in areas undergoing strong mechanical stress, such as the knees and the elbows; it seems that continuous stretching is associated with pro-inflammatory factor production [19]; mechanical stress is also associated with the Koebner phenomenon, appearing not only in psoriasis, but also in vitiligo and lichen planus [20].

There has been substantial research on the specific pathophysiological mechanisms of psoriasis in the last few years. At the level of the skin, non-specific and specific immune cells are recruited to inflammatory foci, which in turn express and secrete inflammatory mediators [21,22]. Vascular endothelial growth factor (VEGF)-associated remodelling of the vascular endothelium, and increased inflammation-linked keratinocyte proliferation have also been detected in immunohistochemical studies from psoriatic patients [23-27]. Since the 1980s, and with the first research efforts specifically focused on elucidating the inflammatory aspects of psoriasis, it has gradually become clear that proinflammatory factors such as interleukins (ILs), tumour necrosis factor α (TNF-α), and interferon γ (IFNγ) play a significant role [28].

The increase in circulating proinflammatory factors in psoriatic patients partially explains the presence of other comorbidities in many such patients; recognition of these comorbidities is important for patient management. It has been documented that there is an increased risk for renal, liver, and cardiovascular (CV) pathologies in patients with active psoriasis [29]. Focusing on the aspect of CV pathologies, psoriasis is an independent risk factor for myocardial infarction; at the same time, atherosclerosis in psoriatic patients is more severe compared with controls [30]. The more severe the psoriasis, the higher the risk of an adverse CV event, such as a myocardial infarction or stroke; it was also found that an increase in psoriasis severity correlated positively with increased levels of aortic inflammation [31]. For each year of active psoriasis, the risk of an adverse cardiovascular event may increase by up to 1% [32]. An increase in the Psoriasis Area and Severity Index (PASI) score will also increase the likelihood of developing metabolic syndrome [33]; this, on its own, is another factor that increases the likelihood of an adverse CV event. Several other comorbidities, frequently appearing in psoriatic patients, may also further increase this likelihood [28].

In the last decades, there has been an increase in available treatment options for psoriatic patients [34], even though the choice of therapeutic agents may be restricted by disease severity or the significant adverse reactions associated with some of them [35,36]. Local treatments are preferred in mild cases but are relatively ineffective in moderate-to-severe cases [37]; systemic treatments are frequently associated with significant side effects, and biological therapies, even if effective, present their own set of challenges [38]. Recent advances in drug design and delivery systems are expected to improve drug efficacy and safety, especially for chronic systemic administration [39]. Based on an increasing corpus of research on phytochemicals [40-42], the role of natural compounds in alleviating psoriasis symptoms is also being explored [43]. Most of these treatment approaches aim to reduce inflammation and oxidative stress, which are hallmarks of psoriasis’s inflammatory nature [28].

While there are specific markers for psoriatic patients that can aid diagnosis, recent research has focused on markers of oxidative stress, inflammation, and hormone levels to assess disease severity and response to therapy. Based on this recent trend, we have decided to examine the levels of several non-specific inflammation markers, i.e., erythrocyte sedimentation rate (ESR), fibrinogen levels, C-reactive protein (CRP), and neutrophil levels in conjunction with the patients’ PASI scores, which are used in the literature to quantify psoriasis severity. We aimed to infer possible associations between fluctuations in the levels of these markers and the severity of psoriasis, and to determine whether, based on their levels, PASI scores can be estimated and vice versa. Given the potential for adverse vascular events in psoriatic patients, we have endeavoured to estimate CV risk for our patients and to propose a predictive model based on our patient data.

## Material and Methods

### Study design

This was an observational cross-sectional study comprising 248 patients with psoriasis vulgaris, selected between May 2022 and October 2024 at the Elias University Hospital, Bucharest. Adult patients (>18 years old) with confirmed clinical and paraclinical data, as defined by internationally accepted criteria, were recruited for the study. Patients with local or systemic infections, other autoimmune conditions, or malignant tumors were excluded from the study. The study was conducted in accordance with the Declaration of Helsinki and was approved by the Ethics Committee of Elias University Hospital, Bucharest.

### Data collection

Collected data included patients’ PASI scores, as evaluated by the attending physician at patient presentation, and inflammation markers, determined by serum analysis and complete blood count (CBC). For the purposes of assessing CV risk, BMI was calculated using the internationally accepted and standardized formula; sex and age were collected from patient files; and smoking status was determined from a structured lifestyle questionnaire.

### Statistical analysis

Statistical analyses were conducted using IBM SPSS Statistics 27. Continuous variables were described by mean ± standard deviation (SD) or median, while categorical variables were described by frequency and percentages. Normality of distribution was assessed using the Kolmogorov–Smirnov and Shapiro–Wilk tests, as well as graphical analysis. The associations between the variables were evaluated using the Spearman coefficient. The group comparisons were performed using independent-samples *t*-tests for normally distributed variables or Mann–Whitney U tests for non-normally distributed variables. The associations between categorical variables were analyzed using the Chi-square test, and the effect sizes were estimated using the Phi coefficient.

Binary logistic regression analyses were used to identify predictors of CV risk. Model fit was assessed using the Hosmer-Lemeshow test and pseudo-R^2^ coefficients (Cox & Snell and Nagelkerke), while collinearity among independent variables was evaluated using tolerance values and variance inflation factors (VIF). Values with *P* < 0.05 were considered statistically significant, corresponding to a confidence level of 95%.

## Results

### Patient demographics and psoriasis severity

In our sample, 50.40% of the patients were women (*n* = 125), and 49.60% were men (*n* = 123). Patient age ranged from 18 to 77 years, with a mean of 48.66 ± 14.08 years and a median of 50 years; no significant age-related differences were observed between sexes.

In the analyzed batch, the PASI score ranged from 1 to 30, with a mean of 8.97 ± 6.48 and a median of 8, and was slightly higher in men (9.33 ± 6.66) than in women (8.62 ± 6.30). Based on the recorded PASI score values, 31.05% of the patients had severe psoriasis (PASI > 10) (*n* = 77), while 68.95% had mild-to-moderate psoriasis (*n* = 171). The frequency of severe psoriasis was higher in men (33.33%) than in women (28.80%).

### ESR values and psoriasis severity

**Figure 1:**
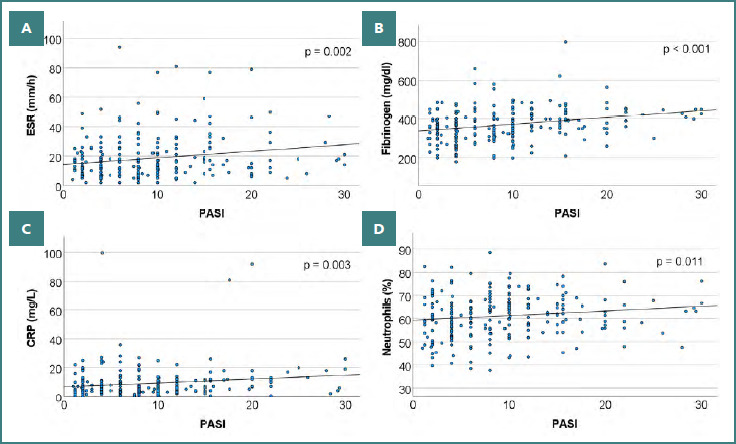
A, Distribution of cases based on PASI score values and ESR levels; B, Distribution of cases based on PASI score values and serum fibrinogen levels; C, Distribution of cases based on PASI score values and serum CRP levels; D, Distribution of cases based on PASI score values and neutrophil count (%); All markers demonstrate statistically significant positive associations with PASI with a general upward trend across increasing disease severity

ESR in general varies with age and gender, and even though each laboratory may have slightly different reference ranges, the maximum values for men are 20 mm/h and for women 30 mm/h, both for individuals over 50 years old [44,45]. In our patients, ESR ranged from 2 mm/h to 94 mm/h, with a mean value of 18.31 mm/h and a median value of 14 mm/h. In 32.26% of patients (*n* = 80), ESR values were increased, while 67.74% (*n* = 168) had values within normal limits (Figure 1A).

The Chi-square test indicated a significant association between ESR (normal vs. increased) and the severity of psoriasis; specifically, 27.49% of patients with mild-to-moderate psoriasis vulgaris (*n* = 47) and 42.86% of those with severe psoriasis (*n* = 33) had increased ESR. The association between psoriasis severity and ESR values was weak (χ^2^ = 5.741, ϕ = 0.152, *P* = 0.017).

In concordance with the aforementioned results, the Mann-Whitney U test showed that patients with severe psoriasis present significantly higher values of ESR (22.91 ± 16.94 mm/h; median 18 mm/h) compared to those with mild-to-moderate form (16.24 ± 13.31 mm/h; median 13 mm/h); the test parameters were U = 8379.5, Z = 3.439, *P* < 0.001. Moreover, the Spearman correlation analysis indicated a positive correlation between the ESR level and the PASI score, treated as a continuous variable, suggesting that higher PASI scores were associated with slightly higher ESR levels (rs = 0.199, *P* = 0.002).

### Fibrinogen levels and psoriasis severity

The physiological reference range for serum fibrinogen in our laboratory is 238-498 mg/dL. In our patients, fibrinogen levels ranged from 180 to 797 mg/dL, with a mean of 369.33 mg/dL and a median of 367 mg/dL. The majority of patients had values within the normal limits (92.34%, *n* = 229), while 4.84% had decreased values (*n* = 12) and 2.82% had increased values (*n* = 7). No significant correlation between psoriasis severity and fibrinogen category (decreased, normal, or increased) was observed (Figure 1B). However, patients with severe psoriasis had a slightly higher frequency of increased fibrinogen (3.90%) than those with mild-to-moderate psoriasis (2.34%).

It should be noted that patients with mild-to-moderate psoriasis exhibited fibrinogen values below the normal range more frequently (5.85%) than those with severe psoriasis (2.60%). In addition, patients with severe psoriasis had fibrinogen values more frequently within normal limits (93.51%) than those with mild-to-moderate psoriasis (91.81%).

The Mann–Whitney U test showed that patients with severe psoriasis presented significantly higher values of fibrinogen (405.77 ± 82.79 mg/dL; median 400 mg/dL) compared to those with mild-to-moderate forms (352.93 ± 78.16 mg/dL; median 350 mg/dL), U = 9214.5, Z = 5.036, *P* < 0.001.

The Spearman correlation indicated a weak-to-moderate direct association between fibrinogen and PASI score, suggesting that greater disease severity is associated with higher fibrinogen levels, even though, in most cases, these values remain within physiological limits (rs = 0.290, *P* < 0.001).

### CRP levels and psoriasis severity

Typically, physiological CRP levels are defined as less than 0.3 mg/dL; the upper limit for normal values in our laboratory is 10 mg/L. In our patients, CRP levels ranged from 0.10 mg/L to 99.70 mg/L, with a mean value of 9.15 mg/L and a median value of 6.00 mg/L; 33.47% (*n* = 83) of the patients presented increased values of CRP, while 66.53% (*n* = 165) of the patients had values within normal limits (Figure 1C).

The Chi-square test indicated a significant association between CRP levels and psoriasis severity. More specifically, 25.73% (*n* = 44) of patients with mild-to-moderate psoriasis and 50.65% (*n* = 39) with severe psoriasis had increased CRP values (χ2 = 14.805, ϕ = 0.244, *P* < 0.001), with an association of weak-to-moderate strength.

Accordingly, the Mann–Whitney U test indicated that patients with severe psoriasis presented significantly higher values of CRP (11.40 ± 13.63 mg/L, median 10 mg/L) compared to those with mild-to-moderate forms (8.14 ± 9.65 mg/L, median 6 mg/L); U = 8301, Z = 3.293, and *P* = 0.001. Moreover, the Spearman correlation analysis indicated a weak positive association between CRP levels and the PASI score (rs = 0.185, P = 0.003), suggesting that higher PASI scores were associated with slightly higher CRP levels.

### Neutrophil levels and psoriasis severity

The percentage of neutrophils ranged from 37.70% to 88.60%, with a mean of 61.09% and a median of 61.45%. The majority of patients (98.39%, *n* = 244) presented values within the normal range, with only 1.6% (*n* = 4) having increased values (Figure 1D).

Compared to patients with mild-to-moderate psoriasis (60.39 ± 9.32 %; median 60%), those with severe psoriasis showed slightly higher neutrophil counts (62.66 ± 8.53 %; median 62.8 %), but the difference did not reach statistical significance (*P* = 0.069).

However, the Spearman correlation analysis indicated a weak positive correlation between neutrophil percentage and PASI score, suggesting that higher disease severity (higher PASI) is associated with a mild increase in neutrophil percentage (rs = 0.161, *P* = 0.011).

### Cardiovascular risk and psoriasis severity: correlations and a predictive model

**Figure 2:**
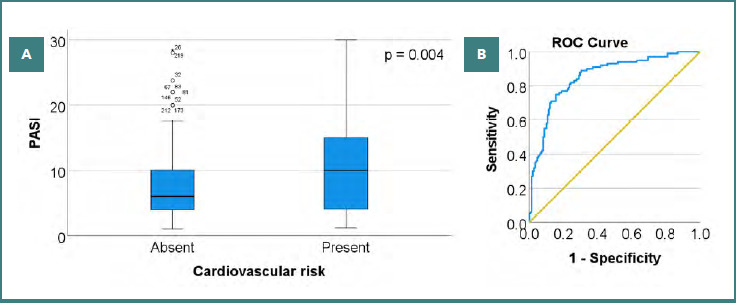
A, Distribution of cardiovascular risk based on patients’ PASI scores, suggesting an association between increased disease severity and cardiovascular comorbidity; individual dots represent outliers; B, The receiver operating characteristic (ROC) curve for our regression model, demonstrating good discriminative ability in distinguishing patients according to cardiovascular risk (PASI = Psoriasis Area and Severity Index).

In the studied batch, 40.32% of patients (*n* = 100) presented CV risk (Figure 2A). It was observed significantly more frequently in patients with severe forms of psoriasis (51.95%, *n* = 40) compared to those with mild-to-moderate forms (35.09%, *n* = 60), the Chi-square test indicating the existence of an association of weak intensity between psoriasis severity and CV risk, χ^2^ = 6.272, ϕ = 0.159, *P* = 0.012. Moreover, the Mann–Whitney U test showed that the PASI score was significantly higher in patients with CV risk (10.49 ± 7.04, median 10) than in those without risk (7.95 ± 5.87, median 6), U = 8981, Z = 2.865, *P* = 0.004.

Binary logistic regression analyses were used to identify predictors of CV risk. Risk prediction was based on a model that included PASI score, sex, age, BMI, and smoking status as indicators of CV risk. The model was statistically significant (χ2 = 102.901, *P* < 0.001) and had moderate explanatory power (Cox & Snell R^2^ = 0.340, Nagelkerke R^2^ = 0.459). The Hosmer-Lemeshow goodness-of-fit test indicated a good fit between the model and the analysed data (χ2 = 10.565, *P* = 0.228). The model classified correctly 79.8% of the cases.

The PASI score was significantly associated with the presence of CV risk (OR = 1.052, CI = 1.000 –1.106; *P* = 0.048). Each 1-point increase in the PASI score was associated with approximately a 5% higher risk of CV events. Age was also significantly associated with CV risk (OR = 1.115, CI = 1.081–1.150; *P* < 0.001), with each additional year of age increasing the odds of CV risk by approximately 11.5%. Also, BMI was significantly associated with the presence of CV risk (OR = 1.105, CI = 1.032–1.183; *P* = 0.004), with each one-unit increase in BMI associated with approximately a 10.5% increase in the odds of CV risk. Sex and smoking were not significant predictors in the multivariate model. The results of the logistic regression analyses indicate that the CV risk of patients with psoriasis is primarily influenced by classic factors such as age and BMI, which have the strongest impact on the model, with disease severity, evaluated via the PASI score, as a predictor of reduced magnitude. The area under the ROC curve (AUC) was 0.854 (CI, 0.806–0.903; *P* < 0.001), indicating good discriminative ability of the model (Figure 2B).

## Discussion

In our study, we endeavoured to gather and analyse data that, based on pathophysiological mechanisms and clinical practice, offer an insight into the link between the intensity of inflammatory processes and psoriasis severity, as evaluated via its cutaneous manifestations and quantified using the PASI score.

Based on our data, there was a significant correlation between psoriasis severity and ESR, and patients with severe psoriasis were more likely to have elevated ESR levels. Several studies have demonstrated that elevated ESR levels are associated with greater disease severity and, consequently, higher PASI scores [46]. Patients with severe psoriasis and high PASI scores tend to have higher ESR levels compared with patients with mild or moderate disease and PASI scores who usually have lower ESR levels [47]. Evidence from psoriatic patients treated with etanercept, methotrexate, and infliximab indicates that PASI score reduction is accompanied by a significant reduction in ESR; this reduction was greater than that observed with other non-specific inflammatory markers [46,48,49]. Therefore, it can be argued that the decrease in ESR reflects the effectiveness of treatment for psoriasis.

Furthermore, there is a documented association between psoriasis and psoriatic arthritis [50, 51]; in the latter case, ESR is considered a good marker for monitoring disease progression [52]. It is therefore conceivable that the same may also be true of psoriasis. Even so, ESR is a non-specific inflammation marker that may also be affected by other ongoing processes [45] and should be interpreted with caution, in conjunction with other biomarkers such as fibrinogen and CRP [46,49].

Regarding fibrinogen, our data showed no significant association between pathologically elevated fibrinogen levels and psoriasis severity, as measured by PASI score. While there was a weak association between psoriasis severity and fibrinogen elevation, most values remained within normal limits. This presents a bit of a conundrum, as psoriasis has a pronounced inflammatory component. On the one hand, fibrinogen is an acute-phase inflammatory marker that reflects systemic inflammation and oxidative stress [49]; on the other hand, it is frequently elevated in patients with chronic inflammatory disorders, such as psoriasis [46]. Based on available research evidence, patients with higher PASI scores have elevated fibrinogen levels, indicating an increased inflammatory and prothrombotic state [46], compared to patients with lower PASI scores; this is in accordance with our findings. On the other hand, fibrinogen elevation in psoriatic patients, even when recorded, does not usually exceed physiological limits, based on our results. Therefore, any upward fluctuations in fibrinogen must be interpreted with caution, regarding its clinical relevance. As with ESR, biological agents such as methotrexate or other systemic treatments usually improve patients’ state, leading to a decrease in PASI score and fibrinogen levels [48]. Moreover, other research indicates that elevated fibrinogen levels in psoriasis patients are associated with an increased risk of CV pathologies, including acute myocardial infarction and stroke, supporting the concept that psoriasis is a systemic disease that affects multiple organs and systems [49,53].

In about one-third of our patients, CRP was elevated above normal limits, and CRP values correlated with disease severity; patients with higher PASI scores were more likely to have elevated CRP. CRP, an acute-phase protein, has been extensively studied in patients with psoriasis [54,55]. Data from the relevant medical literature are consistent with our findings, i.e., that patients with increased psoriasis severity have higher PASI scores [1]; however, in high-sensitivity CRP (hs-CRP) measurements, there was no correlation between hs-CRP and PASI score [56]. On the other hand, in those psoriatic patients, where hs-CRP is elevated, an increased CV risk is documented, again corroborating the systemic nature of the disease [55]. Specialised biological therapies in psoriatic patients, including cyclosporin, methotrexate, and TNF-α antagonists, have been shown to reduce PASI scores and CRP levels. It should be noted that there was a positive correlation between CRP and patient age; in contrast, no such correlation between PASI score values and patient age has been documented [55].

When examining neutrophil levels, only 2% of our patients had an elevated neutrophil count. Neutrophil counts in patients with severe psoriasis were marginally higher than those with mild-to-moderate psoriasis; however, this difference was not statistically significant. Patients with more severe disease may be more likely to have elevated neutrophil counts, but it is unclear whether this slight elevation is actually associated with clinical manifestations. Based on the research by Aktaş Karabay *et al*. [57], neutrophil count and the neutrophil-to-lymphocyte ratio may serve as useful markers of inflammation in psoriatic patients. Neutrophils are the first cells identified in psoriatic plaques, due to an increase of the potent neutrophilic chemotactic factor CXCL8, and high NLR values have been associated with a more severe form of psoriasis and thus a higher PASI score [58-60]. In addition, neutrophilic granules within activated polymorphonuclear neutrophils, when secreted, can activate IL-36, a cytokine that can convert vulgaris psoriasis to a more generalized pustular form and further enhance the inflammatory response [61]. Systemic and biological therapies have been shown to reduce PASI scores, serum neutrophil levels, and NLR values, suggesting their potential role for monitoring disease activity and treatment response [57].

Regarding CV risk in our patients, approximately 40% could be classified as having some form of CV risk, with a higher proportion observed among patients with severe psoriasis. Based on our statistical analysis, there was a weak association between CV risk and psoriasis severity, and PASI scores were significantly higher in patients with CV risk factors. Finally, we attempted to develop a predictive model for increases in CV risk based on PASI scores. In our model, we included the PASI score, sex, age, BMI, and smoking status for each patient. Our model was correct in about 80% of cases when used to classify patients based on CV risk. We determined that for every 1-point increase in PASI score, CV risk increased by about 5%. The association between psoriasis severity and CV risk is based on the chronic, systemic nature of psoriasis. However, its major clinical manifestations primarily involve the skin; the systemic vasculature is also affected [28]. Patients with high PASI scores not only experience more severe skin involvement but also have an increased risk of CVDs, including acute myocardial infarction, stroke, and even premature CV mortality, compared with individuals without psoriasis [58, 60].

This association is driven by persistent systemic inflammation, characterized by highly elevated inflammatory biomarkers, including CRP, fibrinogen, and neutrophils. These inflammatory processes contribute to endothelial dysfunction, atherosclerosis, and other metabolic disturbances. Patients with high PASI scores are associated with a higher prevalence of CV diseases such as systemic arterial hypertension and dyslipidaemia, and are at higher risk for AMI and stroke [55]. Therapy with biological treatments can simultaneously reduce both PASI scores and CV risks associated with psoriasis, highlighting the interplay between skin disease severity and vascular inflammation [55,62].

Despite the interesting conclusions, it is worth noting that our research design and results have several limitations. To start with, our sample size, even if relatively large, was not representative of the Romanian population at a national level; at the same time, some of the results may not be directly translatable at an international level. In addition, in our statistical analysis, we did not assign different weights to values from patients with a familial history of psoriasis. However, not all patients with psoriasis have a strong genetic background; it is reasonable to assume that inflammation markers may be more elevated in patients with a relevant history. Genetic predisposition may increase disease severity or manifest as elevated inflammation markers, even in patients with the same or similar PASI scores. For certain inflammation markers discussed, such as CRP, which may be influenced by prior food or drink consumption, supplements, or medications [63], we did not account for them during sampling. We also have not factored in any lifestyle habits that may aggravate certain inflammation markers, even if they do not directly affect the PASI score. The PASI score, although widely used, has its own limitations, as it has not been fully standardized [64] and may underestimate milder-to-moderate psoriasis, which is the most prevalent form [65]. Dietary constraints or stress levels, which may also influence inflammation status, were not taken into account; certain dietary schemes and increased perceived psychological stress may both exacerbate psoriasis [66]. Irrespective of the particularities of the demographic and lifestyle factors, non-specific inflammation markers are influenced by a variety of factors, especially in patients with comorbidities [67].

Even so, the achieved statistical power was sufficient to detect significant associations for most key variables; the relationship between ESR and CRP, documented in the relevant medical literature, was corroborated by our findings. For fibrinogen and neutrophil levels, no significant associations were observed, but this may be explained by other factors influencing these parameters. Our analysis of the potential to estimate present and future CV risk in our patients indicated that PASI scores can be successfully correlated with the likelihood of adverse CV events, even though other factors, such as age, sex, and BMI, also influence the likelihood.

Regarding recommendations for further research, studies with larger, more diverse population groups, focusing on a variety of specific and non-specific inflammation markers, will be useful for drawing more definite conclusions about the association between inflammation marker levels and systemic inflammation. Given the limitations of the PASI score itself, other scores [68] could be used to provide a better insight into the association between psoriasis severity and inflammation marker levels.

## Conclusion

Non-specific inflammation markers, such as ESR, CRP, serum fibrinogen levels, and neutrophil count, when expressed as percentages of white blood cells, can be useful for evaluating disease activity and severity. Based on our results from a convenience sample representative of the Romanian population, we determined that patients with more severe forms of psoriasis, as evidenced by their PASI scores, had higher ESR, CRP, and serum fibrinogen levels. Patients with more severe forms of the disease were also estimated to have an increased risk for an adverse CV event.
